# Supplementary material for: Modelling Skylarks (Alauda arvensis) to Predict Impacts of Changes in Land Management and Policy: Development and Testing of an Agent-Based Model
Source: PLoS One. 2013 Jun 6;8(6):e65803. doi: 10.1371/journal.pone.0065803 (PMC3675089; doi:10.1371/journal.pone.0065803)
Supplement: Supporting Information S4 — The skylark ODdox as a zipped archive. (ZIP) [file pone.0065803.s004.zip › Skylark_ODdox/class_weather.html]

ALMaSS Skylark ODdox: Weather Class Reference


ALMaSS Skylark ODdox
 2.0


- Main Page
- Related Pages
- Classes
- Files

- Class List
- Class Index
- Class Hierarchy
- Class Members

Public Member Functions |
Private Attributes

Weather Class Reference

`#include <weather.h>`

List of all members.

|  |  |
| --- | --- |
| Public Member Functions | |
| double | GetDDDegs (long a\_date) |
| double | GetGlobalRadiation (long a\_date) |
| double | GetGlobalRadiation (void) |
| double | GetMeanTemp (long a\_date, unsigned int a\_period) |
| double | GetRain (long a\_date) |
| double | GetRain (void) |
| double | GetRainPeriod (long a\_date, unsigned int a\_period) |
| bool | GetSnow (long a\_date) |
| bool | GetSnow (void) |
| double | GetTemp (long a\_date) |
| double | GetTemp (void) |
| double | GetTempPeriod (long a\_date, unsigned int a\_period) |
| double | GetWind (long a\_date) |
| double | GetWind (void) |
| double | GetWindPeriod (long a\_date, unsigned int a\_period) |
| bool | Raining (void) |
| void | Tick (void) |
|  | Weather (const char \*a\_weatherdatafile) |
|  | ~Weather (void) |

|  |  |
| --- | --- |
| Private Attributes | |
| long | m\_datemodulus |
| double | m\_insolation |
| vector< double > | m\_rain |
| bool | m\_rainingtoday |
| double | m\_raintoday |
| int | m\_snow\_decay |
| bool | m\_snowtoday |
| vector< double > | m\_temp |
| double | m\_temptoday |
| vector< double > | m\_wind |
| bool | m\_wind\_valid |
| vector< double > | m\_winddir |
| bool | m\_winddir\_valid |
| double | m\_winddirtoday |
| double | m\_windtoday |

---

## Constructor & Destructor Documentation

|  |  |  |  |  |  |
| --- | --- | --- | --- | --- | --- |
| Weather::Weather | ( | const char \* | *a\_weatherdatafile* | ) |  |

References FloatToDouble(), g\_date, g\_msg, Calendar::GetLastYear(), l\_weather\_starting\_year, m\_datemodulus, m\_rain, m\_snow\_decay, m\_temp, m\_wind, Calendar::Reset(), Calendar::SetFirstYear(), Calendar::SetLastYear(), Tick(), CfgInt::value(), MapErrorMsg::Warn(), and WARN\_FILE.

{

FILE \*ifile;

int Magic, FileFormat, NoDays, Day, Month, Year, FirstYear=0, LastYear=0;

float fTemp, fRain, fWind;

double Temp, Rain, Wind;

//char degree[20];

//double DDegs = 0.0;

ifile = fopen(a\_weatherdatafile, "r" );

if (!ifile) {

g\_msg->Warn(WARN\_FILE, "Weather::Weather(): Unable to open file",

a\_weatherdatafile );

exit(1);

}

fscanf( ifile, "%d", &Magic );

if ( Magic != -1 ) {

// Assume old file format.

NoDays = Magic;

FileFormat = 1;

} else {

fscanf( ifile, "%d %d", &FileFormat, &NoDays );

}

/\*

switch( FileFormat ) {

case 1:

break;

default:

g\_msg->Warn(WARN\_FILE, "Weather::Weather(): "

"Unknown file format specified on line 1: ",

a\_weatherdatafile);

g\_msg->Warn(WARN\_FILE, "First line must be: ",

"-1 <file format specifier> <days worth of weather data>" );

exit(1);

}

\*/

m\_rain.resize( NoDays );

m\_wind.resize( NoDays );

m\_temp.resize( NoDays );

bool storing = false; // Whether we are storing weather data.

unsigned int index = 0;

g\_date->SetLastYear( 0 );

for ( int i=0; i<NoDays; i++) {

// Read in the next data line.

fscanf( ifile, "%d %d %d %g %g %g",

&Year, &Month, &Day, &fTemp, &fWind, &fRain );

FloatToDouble(Temp, fTemp);

FloatToDouble(Rain, fRain);

FloatToDouble(Wind, fWind);

if ( Month == 2 && Day == 29 ) {

// Skip leap days.

continue;

}

if ( Month == 1 && Day == 1 && !storing &&

(

l\_weather\_starting\_year.value() == 0 ||

l\_weather\_starting\_year.value() == Year

)

) {

// Commence storing of data from Jan. 1st of the first

// year requested.

storing = true;

g\_date->SetFirstYear( Year );

FirstYear = Year;

g\_date->Reset();

}

if ( storing ) {

//if (Temp > 0.0 )

// DDegs += Temp;

m\_rain[ index ] = Rain;

m\_wind[ index ] = Wind;

m\_temp[ index ] = Temp;

index++;

}

if ( Month == 12 && Day == 31 && storing ) {

// Found yet another full year worth of weather data.

g\_date->SetLastYear( Year );

LastYear = Year;

//sprintf( degree, "%f", DDegs );

//g\_msg->Warn( WARN\_FILE,

// "Sum of day degrees:",

// degree );

//DDegs = 0.0;

}

}

// Did we find at least one full year worth of data?

if ( g\_date->GetLastYear() == 0 ) {

// Nope...

g\_msg->Warn(WARN\_FILE, "Weather::Weather(): Weather data file did",

"not contain at least one year of data!" );

exit(1);

}

fclose( ifile );

m\_datemodulus = (LastYear - FirstYear + 1)\*365;

m\_snow\_decay = 0;

Tick();

}

|  |  |  |  |  |  |
| --- | --- | --- | --- | --- | --- |
| Weather::~Weather | ( | void |  | ) |  |

{

}

---

## Member Function Documentation

|  |  |  |  |  |  |
| --- | --- | --- | --- | --- | --- |
| double Weather::GetDDDegs | ( | long | *a\_date* | ) |  |

References m\_datemodulus, and m\_temp.

Referenced by VegElement::DoDevelopment(), UnsprayedFieldMargin::DoDevelopment(), and VegElement::SetGrowthPhase().

{

double temp = m\_temp[ a\_date%m\_datemodulus ];

if ( temp < 0.0 ) {

temp = 0.0;

}

return temp;

}

|  |  |  |  |  |  |
| --- | --- | --- | --- | --- | --- |
| double Weather::GetGlobalRadiation | ( | long | *a\_date* | ) |  |

inline

References c\_insolation.

Referenced by Landscape::SupplyGlobalRadiation().

{ return (double) c\_insolation[ a\_date%m\_datemodulus ]; }

|  |  |  |  |  |  |
| --- | --- | --- | --- | --- | --- |
| double Weather::GetGlobalRadiation | ( | void |  | ) |  |

inline

{ return m\_insolation; }

|  |  |  |  |
| --- | --- | --- | --- |
| double Weather::GetMeanTemp | ( | long | *a\_date*, |
|  |  | unsigned int | *a\_period* |
|  | ) |  |  |

References GetTemp().

Referenced by Landscape::SupplyMeanTemp().

{

double sum = 0.0;

for ( unsigned int i=0; i<a\_period; i++ )

sum += GetTemp( a\_date - i );

return sum/(double)a\_period;

}

|  |  |  |  |  |  |
| --- | --- | --- | --- | --- | --- |
| double Weather::GetRain | ( | long | *a\_date* | ) |  |

inline

Referenced by Skylark\_Population\_Manager::DoFirst(), and Landscape::SupplyRain().

{ return m\_rain[ a\_date%m\_datemodulus ]; }

|  |  |  |  |  |  |
| --- | --- | --- | --- | --- | --- |
| double Weather::GetRain | ( | void |  | ) |  |

inline

Referenced by GetRainPeriod().

{ return m\_raintoday; }

|  |  |  |  |
| --- | --- | --- | --- |
| double Weather::GetRainPeriod | ( | long | *a\_date*, |
|  |  | unsigned int | *a\_period* |
|  | ) |  |  |

References GetRain().

Referenced by Farm::BurnStrawStubble(), Farm::CutToHay(), Farm::Harvest(), Farm::HayBailing(), Farm::HayTurning(), Farm::RowCultivation(), Farm::StrawChopping(), Farm::Strigling(), Farm::StriglingSow(), Farm::StubbleHarrowing(), and Landscape::SupplyRainPeriod().

{

double sum = 0.0;

for ( unsigned int i=0; i<a\_period; i++ )

sum += GetRain( a\_date - i );

return sum;

}

|  |  |  |  |  |  |
| --- | --- | --- | --- | --- | --- |
| bool Weather::GetSnow | ( | long | *a\_date* | ) |  |

References Calendar::Date(), g\_date, m\_datemodulus, m\_rain, m\_snowtoday, m\_temp, and random().

Referenced by Landscape::SupplySnowcover().

{

if ( a\_date == g\_date->Date() ) {

return m\_snowtoday;

}

int weatherday = a\_date % m\_datemodulus;

bool snow = false;

if ( m\_temp[ weatherday ] < 0 &&

m\_rain[ weatherday ] > 1 &&

random(100) < 50 ) {

snow = true;

}

// if ( ((dayinyear<90) || (dayinyear>330)) && (rand()%4==1) )

// snow = true;

return snow;

}

|  |  |  |  |  |  |
| --- | --- | --- | --- | --- | --- |
| bool Weather::GetSnow | ( | void |  | ) |  |

inline

{ return m\_snowtoday; }

|  |  |  |  |  |  |
| --- | --- | --- | --- | --- | --- |
| double Weather::GetTemp | ( | long | *a\_date* | ) |  |

inline

Referenced by Farm::FA\_Manure(), Farm::FA\_Sludge(), Farm::FA\_Slurry(), Farm::FP\_Manure(), Farm::FP\_Sludge(), Farm::FP\_Slurry(), and Landscape::SupplyTemp().

{ return m\_temp[ a\_date%m\_datemodulus ]; }

|  |  |  |  |  |  |
| --- | --- | --- | --- | --- | --- |
| double Weather::GetTemp | ( | void |  | ) |  |

inline

Referenced by GetMeanTemp(), and GetTempPeriod().

{ return m\_temptoday; }

|  |  |  |  |
| --- | --- | --- | --- |
| double Weather::GetTempPeriod | ( | long | *a\_date*, |
|  |  | unsigned int | *a\_period* |
|  | ) |  |  |

References GetTemp().

Referenced by Skylark\_Population\_Manager::DoFirst(), and Landscape::SupplyTempPeriod().

{

double sum = 0.0;

for ( unsigned int i=0; i<a\_period; i++ )

sum += GetTemp( a\_date - i );

return sum;

}

|  |  |  |  |  |  |
| --- | --- | --- | --- | --- | --- |
| double Weather::GetWind | ( | long | *a\_date* | ) |  |

inline

Referenced by Farm::FungicideTreat(), Farm::GrowthRegulator(), Farm::HerbicideTreat(), Farm::InsecticideTreat(), Farm::Molluscicide(), Farm::ProductApplication0(), Farm::ProductApplication1(), Landscape::SupplyWind(), and Farm::SynInsecticideTreat().

{ return m\_wind[ a\_date%m\_datemodulus ]; }

|  |  |  |  |  |  |
| --- | --- | --- | --- | --- | --- |
| double Weather::GetWind | ( | void |  | ) |  |

inline

Referenced by GetWindPeriod().

{ return m\_windtoday; }

|  |  |  |  |
| --- | --- | --- | --- |
| double Weather::GetWindPeriod | ( | long | *a\_date*, |
|  |  | unsigned int | *a\_period* |
|  | ) |  |  |

References GetWind().

Referenced by Skylark\_Population\_Manager::DoFirst(), and Landscape::SupplyWindPeriod().

{

double sum = 0.0;

for ( unsigned int i=0; i<a\_period; i++ )

sum += GetWind( a\_date - i );

return sum;

}

|  |  |  |  |  |  |
| --- | --- | --- | --- | --- | --- |
| bool Weather::Raining | ( | void |  | ) |  |

inline

Referenced by Farm::AutumnHarrow(), Farm::AutumnPlough(), Farm::AutumnRoll(), Farm::AutumnSow(), Farm::CutToSilage(), Farm::CutWeeds(), Farm::DeepPlough(), Farm::FA\_AmmoniumSulphate(), Farm::FA\_GreenManure(), Farm::FA\_Manure(), Farm::FA\_NPK(), Farm::FA\_PK(), Farm::FA\_Sludge(), Farm::FA\_Slurry(), Farm::FP\_GreenManure(), Farm::FP\_LiquidNH3(), Farm::FP\_ManganeseSulphate(), Farm::FP\_Manure(), Farm::FP\_NPK(), Farm::FP\_NPKS(), Farm::FP\_PK(), Farm::FP\_Sludge(), Farm::FP\_Slurry(), Farm::FungicideTreat(), Farm::GrowthRegulator(), Farm::HerbicideTreat(), Farm::HillingUp(), Farm::InsecticideTreat(), Farm::Molluscicide(), Farm::ProductApplication0(), Farm::ProductApplication1(), Farm::SpringHarrow(), Farm::SpringPlough(), Farm::SpringRoll(), Farm::SpringSow(), Farm::Swathing(), Farm::SynInsecticideTreat(), Farm::Water(), and Farm::WinterPlough().

{ return m\_rainingtoday; }

|  |  |  |  |  |  |
| --- | --- | --- | --- | --- | --- |
| void Weather::Tick | ( | void |  | ) |  |

References c\_insolation, Calendar::Date(), Calendar::DayInYear(), g\_date, m\_datemodulus, m\_insolation, m\_rain, m\_rainingtoday, m\_raintoday, m\_snow\_decay, m\_snowtoday, m\_temp, m\_temptoday, m\_wind, m\_windtoday, and random().

Referenced by Landscape::Tick(), and Weather().

{

//long dayinyear = g\_date->DayInYear();

int weatherday = g\_date->Date() % m\_datemodulus;

if ( m\_snow\_decay > 0 ) {

m\_snow\_decay--;

}

if ( m\_temp[ weatherday ] > 1.0 ) {

m\_snow\_decay = 0;

}

if ( m\_temp[ weatherday ] < 0.0 &&

m\_rain[ weatherday ] >= 1.0 &&

random(100) < 50 ) {

m\_snow\_decay = (int)(m\_rain[ weatherday ] + 0.5);

}

if ( m\_snow\_decay > 0 ) {

m\_snowtoday = true;

} else {

m\_snowtoday = false;

}

m\_insolation = c\_insolation[g\_date->DayInYear()];

/\*

if ( ((dayinyear<90) || (dayinyear>330)) && (rand()%4==1) )

m\_snowtoday = true;

else

m\_snowtoday = false;

\*/

m\_temptoday = m\_temp[ weatherday ];

m\_raintoday = m\_rain[ weatherday ];

m\_windtoday = m\_wind[ weatherday ];

if ( m\_raintoday > 0.001 )

m\_rainingtoday = true;

else

m\_rainingtoday = false;

}

---

## Member Data Documentation

long Weather::m\_datemodulus

private

Referenced by GetDDDegs(), GetSnow(), Tick(), and Weather().

double Weather::m\_insolation

private

Referenced by Tick().

vector<double> Weather::m\_rain

private

Referenced by GetSnow(), Tick(), and Weather().

bool Weather::m\_rainingtoday

private

Referenced by Tick().

double Weather::m\_raintoday

private

Referenced by Tick().

int Weather::m\_snow\_decay

private

Referenced by Tick(), and Weather().

bool Weather::m\_snowtoday

private

Referenced by GetSnow(), and Tick().

vector<double> Weather::m\_temp

private

Referenced by GetDDDegs(), GetSnow(), Tick(), and Weather().

double Weather::m\_temptoday

private

Referenced by Tick().

vector<double> Weather::m\_wind

private

Referenced by Tick(), and Weather().

bool Weather::m\_wind\_valid

private

vector<double> Weather::m\_winddir

private

bool Weather::m\_winddir\_valid

private

double Weather::m\_winddirtoday

private

double Weather::m\_windtoday

private

Referenced by Tick().

---

The documentation for this class was generated from the following files:

- weather.h
- weather.cpp


- Weather
- Generated on Thu Jan 10 2013 13:15:38 for ALMaSS Skylark ODdox by
   1.8.1.1
